# Description of the genome sequence of *Corynebacterium* species (Marseille-Q4381)

**DOI:** 10.1128/mra.00707-24

**Published:** 2025-01-27

**Authors:** Manon Boxberger, Stéphanie Rivoire, Lorlane Le Targa, Valérie Cenizo, Bernard La Scola

**Affiliations:** 1 UR MEPHI, Aix Marseille University, AP-HM, IHU-Méditerranée Infection, Marseille, France; 2 Groupe L'Occitane, R&D Department, Zone Industrielle Saint Maurice, Manosque, Alpes-de Haute-Provence, France; 3 Biosellal, Dardilly, France; University of California Riverside, Riverside, California, USA

**Keywords:** *Corynebacterium*, skin microbiota

## Abstract

In 2020, we isolated a *Corynebacterium* strain, Marseille-Q4381, from healthy skin. We describe herein its genome sequence and annotation characteristics.

## ANNOUNCEMENT

In 2020, a novel bacterial strain named Marseille-Q4381 was isolated from the forehead skin swab of a healthy 60-year-old woman. Despite efforts, matrix-assisted desorption ionization–time of flight mass spectrometry (MALDI-TOF MS) failed to identify the isolate. Subsequent analysis of the 16S rRNA and genome-to-genome comparison revealed that the taxon belonged to a bacterium within the family Corynebacteriaceae, in the phylum Actinobacteria.

Sampling adhered to the protocol approved by the ethics committee Sud-Est IV (ID-RCB: 2019-A01508-49). Routine subcultures were done on Columbia Agar with 5% sheep blood at 31°C. MALDI-TOF MS protein analysis was conducted using a Microflex spectrometer.

Genomic DNA (gDNA) extraction involved mechanical and chemical pretreatment with 0.5 mm Silica glass beads, acid washed, and a FastPrep-24 5G Grinder, followed by lysozyme incubation and extraction using the EZ1 biorobot with the EZ1 DNA tissues kit (Qiagen). Sequencing and barcoding were performed using MiSeq Technology with the Nextera XT DNA sample prep kit, and read length and paired-end reads were generated from MiSeq. The quality control was conducted using Trimmomatic software v0.36 (1), yielding 959,380 reads before sorting and 922,736 after sorting.

**Fig 1:**
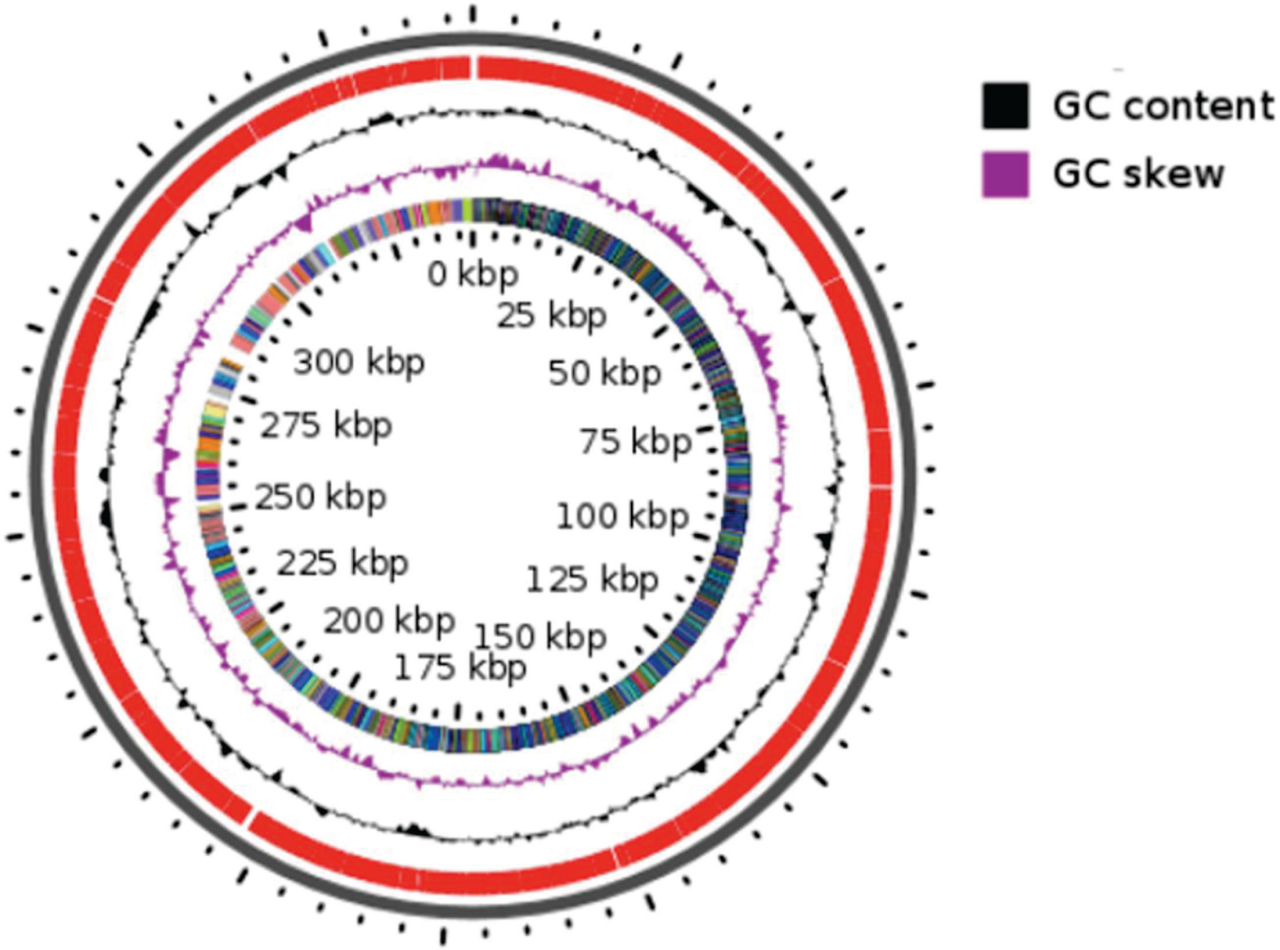
Circular genome map for *Corynebacterium* sp. strain Marseille-Q4381.

Genome assembly with Spades v 3.10 (2) produced 20 contigs with a coverage value of 26.4× and a N50 = 187,76 Kb, Annotation via the PGAP v6,4 pipeline (3) identified 2,112 protein-coding sequences (CDS), the genome contains 60 RNA genes, including 4 rRNAs (1 complete 5S, 1 complete 16S, and 1 complete 23S), with 3 partial 5S rRNAs, 51 tRNAs, and 3 ncRNAs. The circular map of the genome was generated using the CG View server online tool (4). The genome, spanning 2,197,730 base pairs, had a GC content of 65.24% (Fig. 1). All the software tools were used with their default parameters.

To confirm taxonomic position, the 16S rRNA sequence was blasted (BLASTN v2.13.0) against the nr database, and genome sequence data were uploaded to the Type (Strain) Genome Server (TYGS) (5) for taxonomic analysis. Closely related type strains were determined using the MASH algorithm and 16S rRNA gene sequences, with phylogenomic inference conducted through pairwise comparisons and digital DNA-DNA hybridization (dDDH) values calculated. Detailed comparison information is summarized in Table 1.

**TABLE 1:** Comparison between *Corynebacterium* sp. strain Marseille-Q4381 and other relative species^*a*^

Species name	No. Base pairs	Percent G + C	No. proteins	dDDH (d4, in %) compared with *Corynebacterium* sp. strain Marseille-Q4381 genome	C.I. (d4, in %)	G + C content difference (in %) with *Corynebacterium* sp. strain Marseille-Q4381 genome	Bioproject accession reference
*Corynebacterium afermentans* DSM 44280	2,326,687	64.85	2,171	20.7	[18.5–23.1]	0.38	PRJEB18848
*Corynebacterium bouchesdurhonense* SN14	2,255,535	68.02	2,147	21.4	[19.1–23.8]	2.78	PRJEB13138
***Corynebacterium sp strain Marseille-Q4381***	**2,197,730**	**65.24**	**2,120**	**-**	**-**	**-**	PRJNA1077295
*Corynebacterium coyleae* DSM 44184	2,568,936	61.32	2,419	20.7	[18.5–23.1]	3.92	PRJNA303722
*Corynebacterium fournieri* Marseille-P2948	2,357,034	65.04	2,305	21.3	[19.1–23.7]	0.2	PRJEB20393
*Corynebacterium godavarianum* LMG 29598	2,521,298	65.65	2,235	20.9	[18.7–23.3]	0.41	PRJNA555895
*Corynebacterium hadale* NBT06-6	2,679,199	65.18	2,362	20.7	[18.5–23.1]	0.06	PRJNA396693
*Corynebacterium haemomassiliens*e Marseille-Q3615	2,578,128	65.28	2,331	21.2	[18.9–23.6]	0.04	PRJNA646616
*Corynebacterium jeddahense* JCB	2,472,125	67.22	2,341	21.7	[19.4–24.1]	1.98	PRJEB4941
*Corynebacterium mucifaciens* ATCC 700355	2,180,241	65.49	2,028	20.5	[18.3–22.9]	0.25	PRJNA622446
*Corynebacterium riegelii* DSM 44326	2,519,232	60.43	2,283	20.2	[18.0–22.6]	4.81	PRJNA231221
*Corynebacterium senegalense* Marseille-P4329	2,310,902	68.65	2,173	20.5	[18.3–22.9]	3.42	PRJEB24601
*Corynebacterium timonense* 5401744	2,633,085	66.62	2,470	19.7	[17.5–22.1]	1.62	PRJNA303719
*Corynebacterium timonense* DSM 45434	2,551,022	66.86	2,376	19.8	[17.6–22.2]	1.38	PRJEB67
*Corynebacterium tuscaniense* CCUG 51321	2,232,117	59.42	2,073	21.5	[19.3–24.0]	5.82	PRJNA224116
*Corynebacterium ureicelerivorans* DSM 45051	2,328,188	65.01	1,922	21	[18.8–23.4]	0.23	PRJNA257688

^*a*^ Comparing the bacteria to itself is not relevant; the dash ("-") represents with 100%. Bold represents the data of the bacterium.

## Data Availability

The raw sequencing reads have been deposited in the Sequence Read Archive (SRA) under the accession number SRR28047222, and the corresponding BioProject ID is PRJNA1077295. This Whole-Genome Shotgun project has been deposited at DDBJ/ENA/GenBank under the accession number JBAJNR000000000. The version described in this paper is version JBAJNR010000000.
